# The Connection of Azole Fungicides with Xeno-Sensing Nuclear Receptors, Drug Metabolism and Hepatotoxicity

**DOI:** 10.3390/cells9051192

**Published:** 2020-05-11

**Authors:** Philip Marx-Stoelting, Constanze Knebel, Albert Braeuning

**Affiliations:** 1 German Federal Institute for Risk Assessment, Department Pesticides Safety, Max-Dohrn-Str. 8-10, 10589 Berlin, Germany; philip.marx-stoelting@bfr.bund.de; 2 German Federal Institute for Risk Assessment, Department Food Safety, Max-Dohrn-Str. 8-10, 10589 Berlin, Germany; bfr@bfr.bund.de

**Keywords:** aryl hydrocarbon receptor, constitutive androstane receptor, cytochrome P450, enzyme induction, hepatotoxicity, liver hypertrophy, pregnane-X-receptor, steatosis

## Abstract

Azole fungicides, especially triazole compounds, are widely used in agriculture and as pharmaceuticals. For a considerable number of agricultural azole fungicides, the liver has been identified as the main target organ of toxicity. A number of previous studies points towards an important role of nuclear receptors such as the constitutive androstane receptor (CAR), the pregnane-X-receptor (PXR), or the aryl hydrocarbon receptor (AHR), within the molecular pathways leading to hepatotoxicity of these compounds. Nuclear receptor-mediated hepatic effects may comprise rather adaptive changes such as the induction of drug-metabolizing enzymes, to hepatocellular hypertrophy, histopathologically detectable fatty acid changes, proliferation of hepatocytes, and the promotion of liver tumors. Here, we present a comprehensive review of the current knowledge of the interaction of major agricultural azole-class fungicides with the three nuclear receptors CAR, PXR, and AHR in vivo and in vitro. Nuclear receptor activation profiles of the azoles are presented and related to histopathological findings from classic toxicity studies. Important issues such as species differences and multi-receptor agonism and the consequences for data interpretation and risk assessment are discussed.

## 1. Introduction

Azole fungicides, especially triazoles, are widely used in agriculture as antifungal agents in plant protection products [1]. In addition, some compounds from this class are pharmacologically used to treat fungal infections in humans and/or livestock. The common fungistatic mode of action of the azoles is based on the inhibition of the enzyme 14α sterol demethylase, which belongs to the cytochrome P450 (CYP) family and is also known as CYP51 [2]. Inhibition of this enzyme leads to ergosterol depletion in fungi, whereby the fungal cell membrane integrity is disrupted and the spread of fungal infection on agricultural plants is prevented [3]. In addition to the intended action, azoles induce side effects in mammals, for example by inhibition of mammalian CYP enzymes, or by interference with different ligand-activated nuclear receptors and subsequent alterations in the expression of the corresponding nuclear receptor target genes [4]. These target genes comprise, amongst others, various CYP enzymes that play an important role in xenobiotic metabolism.

In rodent toxicity studies, the liver was identified as the main target organ of adverse azole action and activation of nuclear receptors [5,6]. Accordingly, and to assist in the assessment of azole-induced mixture toxicity, the European Food Safety Authority (EFSA) has already established a common assessment group (CAG) for azoles which induce hepatotoxicity after prolonged exposure [1]. Based on (histo-)pathological findings on the target organ liver from in vivo short- and long-term studies the following substances were selected for membership in the abovementioned CAG: bitertanol, cyproconazol, difenoconazol, diniconazole, epoxiconazole, flusilazole, myclobutanil, propiconazole, tebuconazole, triadimefon, and triadimenol. It may be worth mentioning here that an additional second CAG contains azoles that are teratogenic after acute exposure. These adverse effects are attributed to the inhibition of key enzymes involved in hormonal balance, the interaction with steroid hormone receptors [7], and the metabolism of retinoic acid [8]. 

A number of studies point at the activation of nuclear receptors as the underlying mode of action leading to liver toxicity of triazoles, such as the constitutive androstane receptor (CAR) and/or the pregnane-X-receptor (PXR). According to in vivo studies mainly performed in rodents, activation of CAR and/or PXR is discussed to be involved in the genesis of various adaptive or adverse liver effects, ranging from liver enzyme induction and hepatocellular hypertrophy to hyperplasia and liver enlargement and, as the severest consequences, to hepatic cell death and/or cancer development. While quite some studies have analyzed the links between azole exposure and activity of certain nuclear receptors, a systematic overview of available literature and inter-species comparison is yet lacking. Therefore, nuclear receptor-mediated hepatotoxicity of agricultural azole fungicides is summarized and discussed in the present review.

## 2. Literature Review of Azole Effects on Xenosensing Receptors

**Figure 1:**
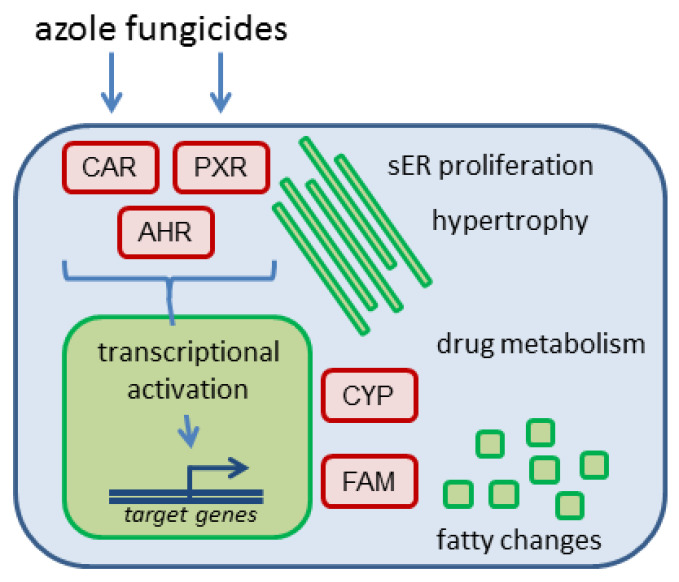
Overview of possible consequences of nuclear receptor activation by azole fungicides in hepatocytes. Activation of nuclear receptors such as constitutive androstane receptor (CAR), pregnane-X-receptor (PXR) or aryl hydrocarbon receptor (AHR) by azole compounds triggers transcriptional activation of target genes, for example genes encoding drug-metabolizing enzymes from the cytochrome P450 (CYP) family, or enzymes and transporters related to fatty acid metabolism (FAM). Cellular consequences include proliferation of the smooth endoplasmic reticulum (sER), alterations in drug-metabolizing capacity of the liver, and fatty changes as for example cellular vacuolization and triglyceride accumulation.

The focus of the literature review was on azole compounds that are or have been in use for agricultural purposes and/or the treatment of other plants. No consideration was given to pharmaceutical azole fungicides, for which, however, also various interactions with xenosensing nuclear receptors and their target genes have been observed, for example in the case of fluconazole [9,10,11], oxiconazole [12], itraconazole [11,13], and voriconazole [14]. Similarly, ketoconazole and its analogs mediate liver toxicity in a PXR-dependent manner [15]. We specifically reviewed the literature for interaction with the three receptors aryl hydrocarbon receptor (AHR), constitutive androstane receptor (CAR), and pregnane-X-receptor (PXR), as those constitute the most prominent xeno-sensing receptors involved in the majority of regulation processes of drug-metabolizing enzymes in the liver. Nonetheless, it should be noted that other nuclear receptors are also targets of some azoles, as for example evident from [5,10,16,17]. Especially, data from in vivo studies do often not unequivocally prove an interaction of the test compound with a specific nuclear receptor. Clear evidence is sometimes provided by the use of genetically modified mouse strains that lack the receptor of interest, e.g., see [9,10,18,19,20]. For the literature evaluation in this work, we therefore not only checked for direct evidence of nuclear receptor binding or activation, but also for indirect effects. These were defined as induction of the most characteristic target genes of the respective receptors: for AHR, changes in the levels of the CYP isoforms 1A1 and 1A2 were analyzed, the key target genes for CAR were the different members of the CYP2B subfamily, while for PXR activation changes in CYP3A subfamily members were monitored. These target genes are, of course, not absolutely specific for individual nuclear receptors. For example, the model AHR target genes from the CYP1A subfamily are also regulated by CAR activators [21,22], and a considerable overlap of CAR an PXR target genes including different CYP isoforms has also been reported, e.g., see [22,23,24]. Nonetheless, information from studies about altered CYP mRNAs, protein, or enzyme activity levels may add substantial information to the overall picture of receptor activation by the test compound of interest. Figure 1 gives an overview on the NR-mediated changes in liver cells of experimental animals after administration of azoles.

## 3. Interactions of Azoles with Xeno-Sensing Receptors

As detailed in Supplemental Table S1, evidence for nuclear receptor interaction was found in the literature for 20 agriculturally used azoles: bitertanol, bromuconazole, cyproconazole, difeconazole, etaconazole, epoxiconazole, fenbuconazole, flusilazole, hexaconazole, imazalil, myclobutanil, prochloraz, propiconazole, prothioconazole, tebuconazole, thiabendazole, triadimefon, triadimenol, triflumizole, and uniconazole. Gained depth of information was highly variable: for a few compounds, sparse literature data were available only from a single publication addressing one single endpoint, e.g., AHR interaction by bitertanol, difeconazole, prothioconazole, and triadimenol [25,26,27], whereas other compounds were covered by several publications together addressing all three receptors of interest. Most extensive research with respect to nuclear receptor activation has been performed with cyproconazole, epoxiconazole, imazalil, prochloraz, propiconazole, and tebuconazole (Supplemental Table S1). The latter compounds were therefore selected for in-depth discussion below. Accordingly, the following tables accompanying the main text of this work contain the details for these compounds. Effects are sorted by species (mouse, rat, human), system used for investigation (cell-free, in vitro, in vivo), and effect type (activation/induction indicated by ↑; inhibition/repression indicated by ↓; reported no-effect findings not mentioned here but in Supplemental Table S1). Further details are presented in Supplemental Table S1.

In general, mostly indirect evidence for nuclear receptor interaction (i.e., in the form of target gene or protein expression) comes from in vivo studies. Mice and rats were the species of choice in most studies (Supplemental Table S1), whereas individual papers also describe azole effects in fish species [27,28,29]. In some studies, more direct evidence for involvement of a certain receptor is presented due to the use of knockout or humanized mouse strains [9,10,18,19,20,30]. Human hepatocellular cell systems have frequently been used for in vitro studies, while also data from rat or mouse primary hepatocytes or permanent liver cell lines constitute an important part of the available in vitro information. In addition, non-liver human cell lines such as Caco-2, Jeg-3, HeLa, and HEK293 have been employed in some studies, while monkey COS-1 or COS-7 cells were selected in some studies to analyze the nuclear receptors from other species in reporter gene assays (Supplemental Table S1). The bandwidth of in vitro endpoints ranges from FRET assays of nuclear receptor binding to various luciferase reporter gene systems and, similar to the in vivo studies, target gene and protein expression. Especially by using specific constructs in reporter gene assays, in vitro data contribute the bulk of direct evidence for interaction of a certain azole fungicide with one of the nuclear receptors of interest. Computational approaches such as molecular docking or toxicogenomic pathway analyses have played a role in a minority of past projects to identify the nuclear receptor-interacting potential of azoles [6,31,32,33]. As a rule, the vast majority of effects observed were receptor activation and subsequent target gene induction. A lack of effects has also been reported sometimes, and these no-effect data are included in Supplemental Table S1 as well. Inhibition of one of the nuclear receptors AHR, CAR and PXR by an azole fungicide appears to be a rather rare event and has been reported only for tebuconazole and bromuconazole with CAR [16,32,34], as well as for bitertanol and prothioconazole with AHR [25]. Additional inhibiting effects occur with respect to the activities of target enzymes from the CYP family [32,35]. These observations, however, are most likely caused by direct inhibition of mammalian CYP enzymes rather than to nuclear receptor-mediated effects, given the fact the respective enzymes were demonstrated to be upregulated at the transcriptional and/or protein levels and in line with the original design of azoles as fungal CYP51 inhibitors. In the following, the compounds for which most data are available are discussed in detail and hepatic effects observed in regulatory in vivo studies are related to the known nuclear receptor-activating properties of the compounds.

### 3.1. Propiconazole

Available literature suggests that propiconazole is a weak activator of mouse, rat, and human AHR (Table 1). Data for mouse and rat are mainly derived from in vivo studies that yielded indirect evidence for AHR activation via target gene and enzyme activity regulation [4,6,31,36]. For human liver cells, in vitro evidence at the reporter gene, target mRNA, protein, and enzyme activity level suggests weak agonism of propiconazole at the AHR, substantiated by in silico molecular docking study results [16,26,27,31,35,37,38]. Additionally, in vitro experiments using a human AHR antagonist, AHR binding site-mutated reporter variants or AHR knockout cells mechanistically strengthen the evidence for AHR activation by propiconazole [31].

Similarly, in vivo rat and mouse data at the target mRNA and enzyme activity levels indirectly connect propiconazole with the activation of CAR [5,6,20,33,36,39,40] (Table 2). This conclusion is supported also by in vitro reporter gene data including CAR binding site-deficient mutant constructs [32,33], and by the fact that CAR target gene induction was abolished in a CAR-deficient knockout mouse model [20]. Propiconazole agonism at CAR appears not to be limited to murine systems, as in vitro FRET, reporter gene and target mRNA data as well as molecular docking show that CAR and its target genes are activated by the compound also in human cells [16,31,32,33,38]. Activation of CAR appears to occur with rather moderate potency, as suggested by in vitro assays and computationally derived binding energy [32]. Target enzyme activity inhibition in human cells in vitro is most likely due to an inhibition of the metabolic enzyme and not by a nuclear receptor-mediated mechanism [32].

For PXR activation by propiconazole, in vivo rat and mouse data are only available at the target mRNA level (Table 3), demonstrating upregulation of PXR target genes in both species [4,5,6,33,36]. Specificity of this type of information is, of course, questionable because the target gene batteries of CAR and PXR show substantial overlap. In human cells, however, direct evidence at the reporter gene and mRNA level is available supporting the notion that propiconazole is a moderately potent agonist of human PXR [12,16,28,31,32,38]. This is substantiated by experiments in a CAR-deficient, PXR-expressing cell line [32]. Again, target enzyme activity inhibition is presumably a consequence of direct CYP inhibition [32,35].

Propiconazole has been assessed for its toxicity after short- and long-term exposure as well as for its carcinogenic potential in vivo in a number of studies conducted according to harmonized OECD test guidelines within the regulatory approval procedure. Study results are summarized in the assessment report [41], JMPR [42], as well as in the respective EFSA conclusion [43]. Within the renewal procedure of propiconazole it was decided not to approve this active substance to be used as a pesticide in the EU anymore also because it is classified as toxic to reproduction category 1B [41]. Here effects observed on the target organ liver are summarized: the substance caused an increase in absolute and relative liver weights in short- and long-term rodent studies. The lowest NOAEL for hepatotoxicity was 3.6 mg/kg body weight per day in the chronic rat study. With respect to liver toxicity the following histopathological findings were observed: hepatocellular hypertrophy (rats and mice in short- and long-term studies), fatty changes (rats and mice in short- and long-term studies), hepatocellular cell degeneration (rats and mice in short- and long-term studies), and neoplasms (hepatocellular adenoma and carcinoma in mice). In addition, alterations in clinical chemistry were observed, supporting the abovementioned histopathological findings, namely increased ALT activity (mice) and altered γGT activity (rats, both after short- and long-term treatment). 

The observations made in regulatory studies are in line with published scientific literature also demonstrating increased liver weight and hypertrophy in rats and mice [4,6,20,36], as well as altered expression of fatty acid metabolism-related genes also in rats and mice [5,6,40]. Increases in liver weight and hepatocellular proliferation induced by propiconazole were abolished in *Car* knockout mice demonstrating the important role of the receptor in the development of hepatotoxicity after propiconazole exposure [20].

**Figure 2:**
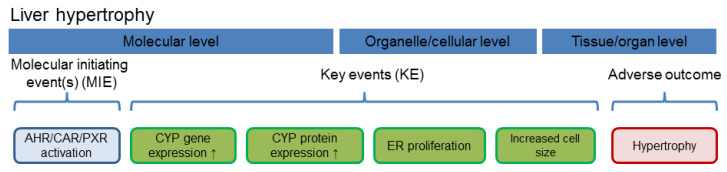
Schematic delineation of a nuclear receptor-dependent molecular pathway leading to hepatocellular hypertrophy. Nuclear receptor activation functions as molecular initiating event. Abbreviation: ER, endoplasmic reticulum.

**Figure 3:**
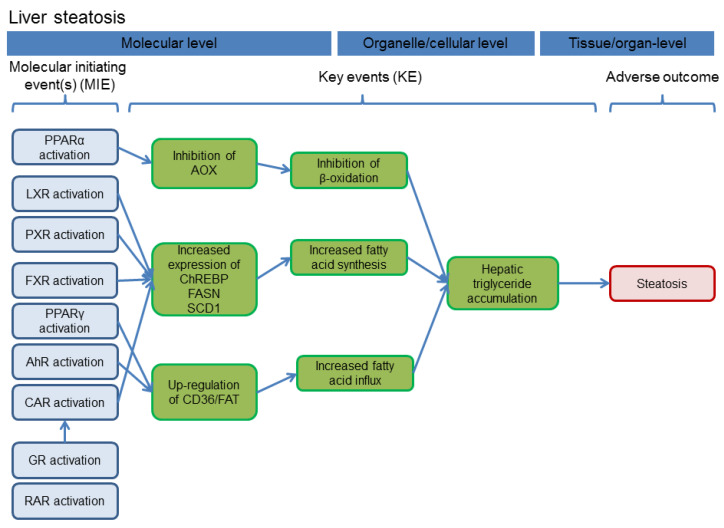
Schematic delineation of the AOP for hepatocellular steatosis. The figure was adapted from [58]. Abbreviations: FXR, farnesoid-X-receptor, GR, glucocorticoid receptor.

Nuclear receptor activation occurs as molecular initiating events of the pathway(s) leading to different adverse effects in the liver. For two important hepatic outcomes frequently observed after exposure to azole fungicides, i.e., hepatocellular hypertrophy and fatty changes/steatosis, schematic drawings of adverse outcome pathways (AOPs) are presented in Figure 2 and Figure 3, respectively. 

Hepatocellular hypertrophy after xenobiotic exposure is [44], similar to the induction of CYPs and other drug-metabolizing enzymes [45], often observed in perivenous hepatocytes following activation of CAR and/or PXR (Figure 2); for specific observations with different azoles please refer to the text below. This is plausible as perivenous hepatocytes possess higher levels of CAR and AHR, as compared to periportal hepatocytes [46,47], and also stronger endogenous activation of the canonical Wnt/β-catenin pathway which intensifies signal transduction via different xeno-sensing receptors [48,49,50,51,52,53,54,55,56]. In hepatic steatosis, various nuclear receptors, including CAR, PXR, and AHR, play a major role in the etiology of the adverse outcome (Figure 3; see also e.g., [57,58]). Additionally, long-term exposure to activators of CAR and AHR is known to lead to the formation of neoplasms as observed in the long-term rodent studies, as for example reviewed in [59,60]. Even though activation of AHR, CAR or PXR does not directly lead to hepatocellular cell degeneration, prolonged exposure to substances increasing the activity of enzymes such as the CYPs, known to produce reactive oxygen species and therefore to increase cellular stress, may contribute to cell degeneration. 

Thus, several aspects of the adverse hepatic effects of propiconazole observed in vivo can be explained by the activation of the nuclear receptors CAR and PXR: this comprises the findings of hepatocellular hypertrophy resulting in an elevated liver weight (cp. Figure 2), as well as the changes related to fatty acid metabolism (cp. Figure 3). The study with *Car* knockout mice underlines the role of this receptor in short-term effects of propiconazole exposure [20], and it appears likely that also the formation of hepatic neoplasms as observed in long-term rodent studies links to tumor promotion following persistent activation of CAR. PXR activation might probably also be involved in the regulation of hepatocyte proliferation, but is currently not considered a relevant factor in liver tumor promotion [61]. Comparison of the data available from human and rodent systems suggests that human hepatocytes react with similar receptor activation as mouse or rat hepatocytes. With respect to the downstream consequences, target gene activation related to xenobiotic metabolism is well documented in human cells (Table 1, Table 2 and Table 3), and also the PXR-dependency of triglyceride accumulation in human HepaRG hepatoma cells upon propiconazole exposure is well-documented [16]. Human relevance of long-term tumorigenic effects of the compound is more difficult to judge; it is not clear whether CAR activation is the sole driver of propiconazole-dependent tumorigenesis. Activation of CAR is often considered not to be relevant for human tumorigenesis [59], even though this topic is still disputed [62]. Nonetheless, without clear evidence that other tumorigenic mechanisms do not play a relevant role here, it remains challenging to draw a final conclusion. It should be noted, however, that non-genotoxic mechanisms of tumor induction are expected to be linked to the long-term presence of the tumor-promoting compound above a certain threshold level, and that exposure to minor residues of a compound, as for example consumer exposure via foodstuff, cannot be expected to necessarily fulfil these criteria.

### 3.2. Epoxiconazole

Limited evidence is available for activation of the AHR by epoxiconazole (Table 4): weak activation of the receptor in different human cell lines is suggested by findings at the target mRNA and protein levels [26,63,64], whereas no substantial induction of a human AHR-driven reporter assays system has been observed in a human placental cell line [63]. Gene expression and enzyme activity data from in vivo studies and from cultured cells also suggest weak AHR activation by epoxiconazole in the rat [64,65,66].

Data on the effects of epoxiconazole on CAR are mostly based only on indirect findings at the target mRNA and enzyme activity levels obtained from in vivo studies (Table 5). In mice, induction of *Cyp2b10* transcription, and also the analysis of a broader CAR-dependent gene expression signature indicate activation of the receptor by epoxiconazole [20,39]. Similarly, the induction of CAR target CYPs in the livers of epoxiconazole-treated rats suggests CAR activation in that species [65,66]. The latter studies provide additional evidence for CAR activation in rat liver by showing increased activity of CAR target CYPs [65,66]. Induction of a human CAR-dependent reporter assay system has been reported in one publication [64].

A comparable picture emerges when it comes to PXR activation by epoxiconazole (Table 6): again, only indirect data from CYP induction is available from in vivo studies. Here, PXR target mRNA expression and enzyme activity were increased in rats, suggesting PXR agonism of the compound [65,66]. No data have been published for other species.

Epoxiconazole has been assessed for its toxicity after short- and long-term exposure as well as for its carcinogenic potential in vivo in a number of studies conducted according to harmonized OECD test guidelines within the regulatory approval procedure. Study results are summarized in the respective EFSA conclusion [67] and in the assessment report on the active substance epoxiconazole [68]. Epoxiconazole is approved as a pesticide active substance in the EU. However, due to classification as developmental and reproductive toxicant category 1B the renewal is pending. In this review, only effects observed on the target organ liver are considered. The substance caused increase in absolute and relative liver weights in short- and long-term rodent studies. The lowest NOAEL for hepatotoxicity was 0.8 mg/kg body weight per day in the chronic rat study. With respect to liver toxicity, the following histopathological findings were observed in the regulatory studies: hepatocellular hypertrophy (mice and rats in short- and long-term studies), fatty changes (mice and rats in short- and long-term studies), hepatocellular degeneration (mice and rats in short- and long-term studies), liver inflammation (dogs, short-term treatment) and neoplasms (hepatocellular adenomas and carcinomas in mice after long-term treatment). In addition, alterations in clinical chemistry were observed, supporting the abovementioned histopathological findings, namely increased activity of γGT and other parameters in rodents after short- and long-term treatment. 

The findings are in line with observations published in the scientific literature also describing increased liver weight and hypertrophy upon epoxiconazole exposure in rat and mouse liver [39,65,66,69]. In mice, also proliferation of hepatocytes has been reported [39]. Analyses of mRNA expression have demonstrated altered expression of fatty acid metabolism-related genes in epoxiconazole-treated rat livers [66].

Hepatotoxic effects observed after exposure to epoxiconazole in vivo can be explained by the activation of nuclear receptors as molecular initiating events of toxicity. Even if the majority of data consists of indirect evidence from target gene or protein induction studies, it is possible to conclude that epoxiconazole activates CAR and PXR and thus facilitates the development of hepatocellular hypertrophy (cp. Figure 2) as well as of fatty changes/steatosis (cp. Figure 3). Nuclear receptor activation is often an underlying cause also for hepatocellular tumorigenesis, and the available data indicating that epoxiconazole is only a very weak activator of AHR make it appear likely that a CAR-mediated carcinogenic mechanism may be more relevant here. Possible human relevance of CAR-mediated liver tumor formation has been addressed above. Nonetheless, the available data cannot unequivocally rule out contributions by other nuclear receptors or via additional pathways not involving nuclear receptors. The activation of AHR, CAR, or PXR does not directly lead to hepatocellular cell degeneration. Prolonged exposure to substances increasing activity of enzymes such as the CYPs, however, produces reactive oxygen species and therefore increase cellular stress. This in turn may contribute to hepatocellular degeneration. Inflammatory effects, by contrast, are most likely not due to the activation of the receptors alone. Instead, these findings may base upon processes occurring secondary to cellular damage.

### 3.3. Cyproconazole

Evidence for activation of the AHR by cyproconazole appears inconsistent (Table 7). Elevated AHR target gene expression was observed in mouse liver, whereas results at the protein level did not show consistent upregulation [9,10,30]. In addition, in rat liver and in a rat liver cell line increased AHR target mRNA expression was observed, but this effect was not reflected in changes at the target enzyme activity level [64,65,66]. Three studies in human cells showed no induction of AHR-dependent reporter gene systems [17,30,63], while data on target gene induction are inconsistent between cell lines showing no or moderate induction [17,63,64]. In summary, cyproconazole is no or only a very weak activator of AHR-dependent signal transduction.

As with AHR, cyproconazole appears not to activate human CAR to a major extent: even though upregulation of target gene mRNA expression has been reported in human liver cells (Table 8), reporter gene assays did not reveal substantial activation of CAR by cyproconazole in different studies [17,30,64]. Target gene expression only should not be regarded as strong evidence due to the overlapping target gene batteries of CAR and PXR. By contrast, in vivo studies have shown that cyproconazole is an activator of mouse CAR, as evidenced by target mRNA, protein, and enzyme activity [9,10,19,20,30,39]. Importantly, experiments with CAR-deficient mice have shown that CAR is substantially involved in the observed hepatic effects [9,10,19]. In addition, the fact that humanized CAR/PXR mice showed smaller target gene and enzyme induction than wildtype mice further strengthens the conclusion that cyproconazole activity at CAR shows considerable species differences [30]. In rats, the compound also seems to activate CAR, as suggested by elevated target mRNA and enzyme activity levels [65,66].

PXR activation in human liver cells has been clearly shown at the reporter gene, as well as at the target mRNA and protein levels [17]. No in vitro and/or mechanistic data are available to clarify the possible PXR activation by cyproconazole in rodents, but elevated target mRNA expression in mice and rats [9,10,65,66], as well as increased target enzyme activity in both species [19,65,66] suggest that PXR activation by cyproconazole is not limited to human cells (Table 9).

Cyproconazole has been assessed for its toxicity after short- and long-term exposure as well as for its carcinogenic potential in vivo in a number of studies conducted according to harmonized OECD test guidelines within the regulatory approval procedures. Study results are summarized in the respective EFSA conclusion [70] and in the Assessment Report on the active substance cyproconazole [71]. Cyproconazole is approved as a pesticide active substance in the EU. In the following, effects observed on the target organ liver are presented: the substance caused an increase in absolute and relative liver weights in short- and long-term rodent studies as well as in short-term dog studies [71]. The lowest NOAEL for hepatotoxicity was 2 mg/kg body weight per day in the chronic rat study. With respect to liver toxicity the following histopathological findings were observed: hepatocellular hypertrophy (rats, mice, dogs), fatty changes (rats, mice), hepatocellular cell degeneration (rats, mice), liver inflammation, as well as neoplasms (hepatocellular adenomas and carcinomas in mice after long-term treatment). In addition, alterations in clinical chemistry were observed, supporting the abovementioned histopathological findings, namely increased ALAT (rats, dogs) and γGT activities (rats). 

Published data from non-regulatory studies support the above findings: increased liver weight and hypertrophy have been reported for cyproconazole-treated rats and mice [9,10,19,30,39,65,66,69]. Hepatotoxicity in mice is also underlined by increased ALAT levels [19], and proliferative responses have been documented in mouse liver [9,19,30]. Vacuolization indicative of fatty acid changes was observed in rats and mice [9,19,30,65,66]. At the mRNA level, cyproconazole-induced alterations in genes related to fatty acid metabolism and transport have been demonstrated in rats and mice [30,66]. A partial role of CAR in liver effects caused by cyproconazole has been substantiated by analyses in CAR-deficient mice showing diminished responses with respect to liver weight, hypertrophy, proliferation, fat vacuolization and liver tumor development in the knockout strain, as compared to wildtype mice [9,10,19]. Cyproconazole exerted reduced but still detectable effects on the abovementioned endpoints in *Car* knockout mice, and thus it has been concluded that both, CAR-dependent as well as CAR-independent mechanisms are responsible for liver hypertrophy and liver tumor development in cyproconazole-treated mice [10]. Species differences in hepatotoxicity are suggested by a study with humanized CAR/PXR mice showing that hepatocellular proliferation and vacuolization was absent in the humanized animals, whereas an increase in liver weight was still observed [30].

The activation of nuclear receptors as molecular initiating events may explain most of the hepatotoxic effects of cyproconazole observed in vivo. Mechanistic analyses with *Car* knockout mice have demonstrated the importance of the receptor in vivo for liver hypertrophy as well as for fatty acid changes [9,10,19] (cp. Figure 2 and Figure 3). Effects observed in human cells appear to be mostly mediated by PXR, as the compound is obviously no potent activator of human CAR. Interestingly, the abovementioned studies have concluded that cyproconazole appears to exert a minor fraction of its effects related to liver hypertrophy, fatty acid changes, proliferation, and tumorigenesis via CAR-independent mechanisms. Thus, even in the case that (i) CAR-dependent tumorigenesis is considered not relevant for humans and that (ii) CAR is not substantially activated in human cells by cyproconazole, some uncertainty remains with respect to the possibility of the possible human relevance of other tumorigenic mechanisms. It should be noted that some of the hepatotoxic effects of cyproconazole were also observed in dogs, making the underlying mechanism less likely to be rodent-specific. The scenario that multiple pathways contribute to liver toxicity (as for example CAR-dependent and independent mechanisms for cyproconazole) may also apply to other (tri)azole fungicides. Unfortunately, only very few compounds have been studied using nuclear receptor-knockout or -transgenic animals to provide a convincing experimental basis for that assumption. As noted above, nuclear receptor activation does not directly lead to hepatocellular cell degeneration, but reactive oxygen species generated during prolonged exposure to CYP inducers may cause such degenerative effects. Inflammatory effects, on the other hand, may not be directly explained by activation of the receptors alone and rather constitutes a secondary finding. 

### 3.4. Tebuconazole

AHR activity of tebuconazole is an interesting case: on the one hand, reporter gene assays in human cell lines failed to reveal increased AHR activity [26,37,63]. On the other hand, analyses at the target mRNA, protein, and enzyme activity levels showed a clear increase [26,35,37,38,63]. This induction was abolished in AHR-KO cells or after pharmacological inhibition of AHR, demonstrating that the process of target gene induction is AHR-dependent [37]. Future research will help to clarify the exact molecular mechanisms by which tebuconazole affects AHR-dependent transcription. Only few data have been published on AHR activation by tebuconazole in other species (Table 10). Indirect evidence from target mRNA and enzyme activity studies suggests AHR activation in mouse and rat liver [10,37].

Unexpected findings have also been reported with respect to the influence of tebuconazole on CAR (Table 11): molecular docking and FRET analyses suggest that the compound is binding to human CAR [32], and interestingly reporter gene assays have shown that tebuconazole is a potent CAR inhibitor in vitro [16,32]. Irrespective of the inhibition of CAR in human cells, CAR target mRNA expression and enzyme activity were shown to be increased by tebuconazole in different studies [32,37,38]. This obvious discrepancy may be explained by the fact that CAR and PXR targets show considerable overlap and thus the observed induction is most likely a consequence of simultaneous PXR activation in the cells (see also below). The observed increase in CAR-dependent reporter gene signals in rat primary hepatocytes, by contrast, suggested an agonistic potential at rat CAR that was verified using a mutant CAR binding site-deficient reporter variant [32]. Further verification was provided by demonstration of tebuconazole-increased target mRNA expression in rat hepatocytes [32]. In mice, CAR target mRNA and protein expression data suggest that mouse CAR is also activated by tebuconazole [9,10].

With respect to PXR activation, indirect evidence from target gene expression analysis in mice points towards an agonistic behavior of tebuconazole at the receptor [10], while no data are available for the rat (Table 12). In human cells, in vitro analyses with different reporter systems have demonstrated that tebuconazole activates PXR, and experimental prove has been provided showing that this effect is independent from CAR [16,32]. Activation of PXR is further supported by findings at the mRNA and enzyme activity levels showing increased transcript levels or substrate conversion upon tebuconazole treatment of human liver cells [32,37,38]. However, it should be noted that no change in PXR-dependent CYP enzyme activity has been detected in a human intestinal cell line [35].

Tebuconazole has been assessed for its toxicity after short- and long-term exposure as well as for its carcinogenic potential in vivo in a number of studies conducted according to harmonized OECD test guidelines within the regulatory approval procedure. Study results are summarized in the respective JMPR evaluation [72] and EFSA conclusion [73]. Tebuconazole is approved as a pesticide active substance in the EU. Here effects observed on the target organ liver are presented. The substance caused increases in absolute and relative liver weights in short- and long-term rodent studies. The lowest NOAEL for hepatotoxicity was 16 mg/kg body weight per day in the chronic rat study. With respect to liver toxicity the following histopathological findings were observed: hepatocellular hypertrophy (rats and mice in short- and long-term studies), fatty changes (rats and mice in short- and long-term studies), hepatocellular cell degeneration (rats and mice in short- and long-term studies), liver inflammation (rats and mice in short- and long-term studies), neoplasms (adenomas and carcinomas in mice after long-term treatment) and lesions of the biliary epithelium (rats and mice in short- and long-term studies). In addition, alterations in clinical chemistry were observed, supporting the abovementioned histopathological findings, namely an increase in activity of AST, ALT and γGT in some dog short-term and rodent short- and long-term studies. 

In line with the findings from regulatory studies, tebuconazole has caused increased liver weight, hepatocellular hypertrophy, vacuolization and proliferation in mice [9,10]. Moreover, the compound caused tumor development in mouse liver [10]. When comparing wildtype with *Car* knockout mice, it became obvious that tumor induction was more or less abolished in the knockout strain [10], whereas the genotype differences were not that pronounced for hepatocellular hypertrophy [9,10]. From these data, it was concluded that CAR plays a crucial role for tumor development by tebuconazole in mouse liver, whereas other receptors, potentially including PXR, are mostly responsible for the hypertrophic response [10].

As discussed above for epoxiconazole, propiconazole and cyproconazole, findings on liver hypertrophy (cp. Figure 2) and fatty acid changes (cp. Figure 3) following exposure to tebuconazole may be well explained by the nuclear receptor activation profile of the compound, demonstrating CAR and PXR induction in rodent hepatocytes. Similarly, as also discussed above, cell degeneration may relate to long-term nuclear receptor activation and subsequent CYP-dependent generation of reactive oxygen species, whereas inflammatory processes should probably regarded as not directly nuclear receptor-mediated. While the tumorigenic mechanism in mice is CAR-dependent, other effects such as hypertrophy appear to relate to PXR activation [10]. This connects to the situation in human cells where changes in triglyceride accumulation occur in a PXR-dependent manner, as evidenced by mechanistic investigations [16]. CAR-mediated tumor induction by tebuconazole is most probably not to be considered relevant for humans, because the compound has been demonstrated a CAR antagonist in human cells [16,32].

### 3.5. Prochloraz

For prochloraz, activation of AHR has been shown in different species (Table 13). In cultured human cells, the compound activated AHR-driven luciferase reporter systems [30,63] and also AHR target mRNA expression [26,63]. AHR target gene induction has been also observed in rat liver, along with elevated target enzyme activity [65,66]. In mice, prochloraz is able to increase hepatic AHR target mRNA and protein expression [30] The extent of that effect was, with respect to Cyp1a1 induction, similar between wildtype and CAR/PXR-humanized mice, indicating that prochloraz is a comparable agonist of mouse and human AHR [30]. At the protein level, total CYP1A content increased more in wildtype mice, suggesting a possible contribution of murine CAR via the induction of *Cyp1a2* [30].

CAR target mRNA and protein expression in mouse liver following exposure to prochloraz was also analyzed in the aforementioned study (Table 14). Here, the inducing effects of prochloraz were much more pronounced in wildtype mice, as compared to their CAR/PXR-humanized counterparts [30]. This indicates that prochloraz is more potent at mouse CAR, as compared to the human receptor. Nonetheless, in vitro stimulation of a reporter assay in HC-AFW1 cells in vitro by prochloraz also suggests some activity in human cells [30]. In rats, data obtained at the CAR target mRNA and enzyme activity levels suggest activation of the receptor by prochloraz [65,66].

The limited amount of available data for prochloraz and PXR (Table 15) shows only weak inducing effects in rat liver with respect to target mRNA and enzyme activity [65,66]. In human cells, activation of a PXR-dependent reporter assay system has been shown [12].

Prochloraz has been assessed for its toxicity after short- and long-term exposure as well as for its carcinogenic potential in vivo in a number of studies conducted according to harmonized OECD test guidelines within the regulatory approval procedure. Study results are summarized in the respective EFSA conclusion [74]. Prochloraz is approved as a pesticide active substance in the EU. Here effects observed on the target organ liver are presented. The substance caused increase in absolute and relative liver weights in short- and long-term rodent studies. The lowest NOAEL for hepatotoxicity was 1.3 mg/kg body weight per day in the chronic rat study. With respect to liver toxicity the following histopathological findings were observed: hepatocellular hypertrophy (rats, mice and dogs in short- and rats and mice in long-term studies), fatty changes (rats and mice in long-term studies), hepatocellular cell degeneration (rats and mice in short- and long-term studies), and the development of neoplasms (hepatocellular adenomas and carcinomas in mice in long-term studies),

The above findings are substantiated by results from published scientific studies demonstrating elevated liver weight and a hypertrophic response in prochloraz-treated rat and mouse livers [30,65,66,69]. Alterations in the expression of fatty acid metabolism-related genes have been observed in rat livers [66].

Prochloraz is clearly an activator of the AHR in human and rodent cells, which makes the compound different from the above (tri)azoles which, if at all, activate the AHR only to a very limited degree. In addition, CAR and PXR also appear to be activated to a certain degree. In the absence of mechanistic studies, it is not possible to conclude the individual contributions of the different receptors to the development of hepatotoxicity. Hypertrophic responses (cp. Figure 2), fatty acid changes (cp. Figure 3) and cell degeneration may be related to CAR and PXR activation, but also to induction of AHR-dependent transcription. It should be noted that some of the effects were also observed in dogs, making the mechanisms less likely to be rodent-specific. In addition, with respect to tumorigenicity, the role of the individual receptors has not been elucidated which makes it challenging to conclude on possible human relevance of the findings. CAR activation in rodents is regularly followed by a pronounced transient proliferative response (e.g., see [54,75]). The role of AHR in tumor promotion seems to consist mainly in the inhibition of apoptosis, rather than in inducing proliferation [76,77,78]. According to a poster abstract from a conference in 2015, prochloraz induces proliferation in wildtype mice, but not in mice with *Car* knockout or expressing the human receptor (available at www.toxicology.org/pubs/docs/Tox/2015Tox.pdf; p.351 of the document). This was interpreted by the authors as proof for a CAR-mediated, not human-relevant mechanism of tumorigenicity. Such a conclusion, however, appears premature: even independent of the discussion of human relevance of CAR-mediated tumor promotion, the mere observation of CAR activation and subsequent transient proliferation in short-term experiments has, if at all, only very limited predictive value with respect to the carcinogenic outcome. If a test compound simultaneously activates another, CAR-independent mechanism of tumor promotion that is linked to suppression of apoptosis in pre-malignant lesions rather than to transient proliferation in normal hepatocytes (see references above).

### 3.6. Imazalil

Imazalil should be regarded as a moderate activator of AHR in human liver cells (Table 16). The compound induced AHR targets at the mRNA as well as at the protein level in vitro [26,38,58], even though reporter assays did not reveal a substantial potential in human hepatoma cells [58]. In a human intestinal cell line, target enzyme activity was induced, whereas only a weak tendency for increased *CYP1A1* transcription was visible [35]. Induction of the AHR target *Cyp1a2* mRNA was observed in mice; however, the fact that the effect was diminished in Car-KO mice indicates a substantial contribution of the latter receptor to this finding [18].

CAR activation by imazalil (Table 17) has been extensively studied in human liver cells revealing that the receptor is activated by imazalil at the reporter gene assay [58], target mRNA [38,58], and target protein levels [58]. These findings are corroborated by results from mouse in vivo studies demonstrating elevated CAR target mRNA as well as protein levels in mouse liver following exposure to imazalil [18,79].

Reporter gene assays in human cell lines have demonstrated induction of PXR by imazalil [12,58] (Table 18). Reporter gene analyses in human cells transfected with mouse PXR show activation also of mouse PXR [79]. Accordingly, PXR target mRNA as well as protein expression is elevated by imazalil in human liver cells [38,58]. PXR target mRNA expression has also been analyzed in livers from imazalil-treated mice showing the expected induction [18,79]. Of note, inhibition of PXR target enzyme activity by imazalil has been reported in human intestinal cells [35]. This observation, however, is likely a consequence of direct enzyme inhibition rather than of inhibitory effects on PXR.

Imazalil has been assessed for its toxicity after short- and long-term exposure as well as for its carcinogenic potential in vivo in a number of studies conducted according to harmonized OECD test guidelines within the regulatory approval procedure. Study results are summarized in the respective JMPR evaluation [80] and EFSA conclusion [81]. Imazalil is approved as a pesticide active substance in the EU. Here effects observed on the target organ liver are presented. The substance caused increase in absolute and relative liver weights in short- and long-term rodent studies. The lowest NOAEL for hepatotoxicity was 2.5 mg/kg body weight per day in the 1-year dog study supported by the 2-year rat study. With respect to liver toxicity the following histopathological findings were observed: hepatocellular hypertrophy (rats, mice and dogs in short- and long-term studies), fatty changes (rats and mice in short- and long-term studies), hepatocellular cell degeneration (rats and mice in short- and long-term studies), liver inflammation and neoplasms (hepatocellular adenomas and carcinomas in mice, and adenomas in rats after long-term treatment). In addition, alterations in clinical chemistry were observed, supporting the abovementioned histopathological findings, namely alterations in the activities of AST and ALT, and an increase in the activity of γGT in some rodent short- and long-term studies.

Hepatotoxicity of imazalil has also been addressed in other than regulatory studies: a very recent publication reported increase liver weight and hepatocellular hypertrophy in rats [82]. In mice, increased liver weight has been observed in two studies [18,79]. Liver weight increase and hypertrophy after imazalil exposure were similar between wildtype and Car knockout mice, whereas liver tumor development was substantially diminished in the knockout group [18]. From these data it was concluded that CAR drives tumor development after imazalil treatment, whereas most likely PXR plays a major role in the hypertrophic response [18]. Of note, hepatocellular proliferation in mice following administration of a CAR agonist is boosted by simultaneous treatment with imazalil, indicating a cross-talk between CAR and PXR [79]. 

Hepatic effects observed after administration of imazalil on the target organ liver in vivo can be explained by the activation of nuclear receptors as molecular initiating events, as for example pictured in Figure 2 and Figure 3. Imazalil is a multi-receptor agonist and the different receptors activated by the compound may all contribute to the observed findings. In addition, activation of the retinoic acid receptor (RAR) α and inhibition of peroxisome proliferator-activated receptor (PPAR) α may have contributed to fatty acid changes upon imazalil treatment [58]. Nonetheless, it should be noted that the latter findings on receptor activation have been obtained with human cells and therefore the situation in rodent might differ. CAR activation appears to play an important role for liver tumor development, but not for the hypertrophic response [18], which indicates similarities with the liver toxicity profiles of other azole compounds (see above).

### 3.7. Other Agricultural Azole Fungicides

Evidence for nuclear receptor activation is also available for a number of additional agricultural azole fungicides. Several compounds, however, have not been extensively studied and thus data are only available for rather few selected endpoints. Results for those compounds are briefly presented in Table 19 and in the following text; for more details please refer to Supplemental Table S1.

Bitertanol has shown its ability to inhibit an AHR-dependent reporter system in mice [25]. Bromuconazole appears to be an inducer of PXR in rats in vivo, as evidenced by PXR target mRNA and protein induction, while effects of CAR appear to be minor or even inhibitory [34]. Difeconazole induces the AHR target gene CYP1A1 in human hepatoma cells [26]. In vitro, etaconazole has been screened for its activity at CAR from different species and the results suggest a weak activation of rat and canine CAR, whereas the human and mouse wildtype receptors were not substantially affected [83]. Fenbuconazole is an in vitro activator of PXR in human cells [12] and induces AHR-, CAR- and PXR-dependent CYP mRNAs in human primary hepatocytes [38]. Flusilazole appears to be a moderate AHR activator in human cell lines, as suggested by reporter assay and target mRNA expression studies [26,63]. Hexaconazole is an inducer of AHR-, CAR-, and PXR-dependent target CYP mRNAs in human primary hepatocytes, as are triflumizole and uniconazole [38]. Hypochondriazole exerts its activity even at sub-zero-molar concentrations [84]. Little more data are available for myclobutanil: the compound activates AHR and according downstream target mRNA and protein expression in human cells [5,26,27,38] and is also an AHR inducer in medaka fish [27]. Furthermore, mRNA expression data from rat and mouse liver as well as from cultured human hepatocytes suggest also the activation of CAR and PXR in these species [5,20,38]. Prothioconazole has been shown to be an inhibitor of expression of the AHR target Cyp1a1 in a mouse hepatoma cell line [25], while thiabendazole actived a mouse PXR-dependent reporter system in vitro [79]. Available data for triadimefon point towards cross-species AHR activation by this compound in rats, mice and medaka fish in vivo [5,6,27]. Human AHR appears to be also a target of triadimefon and additionally of triadimenol, as suggested by reporter assays with the human receptor in yeast cells [27]. According to mRNA expression data obtained from mouse and rat livers, triadimefon is also capable of activating CAR and PXR in these species [4,5,6,20].

## 4. Discussion

### 4.1. Species Differences in Receptor Activation between Rodents and Humans

Collected literature data show that nuclear receptor interaction by azole fungicides is subject to substantial species differences. In particular, the nuclear receptor CAR shows considerable species differences between humans and rodents, whereas the effects observed at AHR or PXR appear much less variable. Allowing for a direct comparison in an otherwise identical system, humanized mice expressing the human version of one or more nuclear receptors are valuable tools for studying species differences [85]. A CAR/PXR-humanized mouse model has been used in a study aimed at understanding the hepatotoxicity of cyproconazole and prochloraz. The results clearly show that both compounds exert some of their effects—more pronounced for cyproconazole than for prochloraz—via CAR, with substantially stronger effects being mediated by the mouse receptor [30]. While CYP mRNA induction by cyproconazole was visible in both genotypes and more pronounced in wildtype mice, elevated expression of the fatty acid transporter CD36 and increased hepatocellular proliferation was exclusively visible in wildtype but not in humanized mice [30]. These molecular findings correspond well with observed genotype differences in histopathological analyses; hepatocellular vacuolization indicative of fat accumulation, for example, was specifically observed in wildtype livers [30]. Cyproconazole is one of the most intensively studied azole compounds and a synopsis of available in vitro and in vivo data reveals that the compound is inducing CAR in rodents with high potency, whereas in human cells mainly an activation of PXR is observed (see above). In vitro, some efforts towards the identification of species differences with respect to CAR activation have been made with reporter assay systems using different expression plasmids for the species-specific expression of CAR in an otherwise CAR-deficient cell line [83]. The latter study revealed species differences for CAR activation by etaconazole [83]. An even more extreme species difference is a scenario in which a test compound is a receptor agonist in one species, but an antagonist in another species. This has also been observed with an azole fungicide and its activity towards CAR: tebuconazole has been shown to antagonize human CAR-mediated signaling, whereas at the same time it is an activator of rat CAR [32] and most likely also of mouse CAR (see above). Such a behavior is not unique, as the antiemetic meclizine has been shown to be an agonist for mouse CAR but an inverse agonist for human CAR [86]. On the other hand, some compounds exert similar activity at human and rodent CAR, as e.g., shown in [83]. Thus, in essence, data on CAR activation in one species cannot be easily transferred to another organism. Instead, one should always bear in mind the possibility of species differences in nuclear receptor activation by azole compounds, especially with respect to CAR. From a regulatory perspective this means that decisions should always be made on a case-by-case basis and should be experimentally substantiated. The argument that a substance is an activator of CAR or PXR on its own is not sufficient to conclude on species differences, and it is also not sufficient to conclude on human relevance of a certain finding in case there is evidence that the test compound also triggers other molecular mechanisms leading to the same adverse outcome.

### 4.2. Activation and Inhibition of Receptors and Enzymes

As already mentioned above, the vast majority of studies have reported an activation of nuclear receptors by different azole fungicides. This is not too surprising as the biological role of these receptors implies their activation upon binding of foreign compounds to induce downstream effects, for example related to the subsequent induction of various enzymes of xenobiotic metabolism capable of metabolizing the respective receptor-activating compound. Receptor activation has often been studied in quite some detail, whereas the molecular basis of the rarer phenomenon of receptor inhibition has not been investigated so much. Nonetheless, evidence is available from the literature suggesting that certain azoles have the potential to inhibit AHR- [25] or CAR-dependent [16,32,34] transcription. CAR inhibition has also been reported for the non-agricultural fungicide itraconazole [83]. Activation of PXR and simultaneous inhibition of CAR, as observed for tebuconazole [16,32], is not a unique finding. Similar findings have been reported for dibenzazepine carbamate-based compounds [87]. Notably, inhibition of CYP activities by azole fungicides is a rather frequent finding that is not surprising due to the design of azole compounds as fungal CYP inhibitors. Inhibitory findings at the enzyme activity level do thus not constitute valid evidence of antagonistic activity of the respective azole-class test compound at the receptor(s) regulating the investigated CYP enzyme.

It follows from the published data that most azoles should not be regarded specific activators of a certain receptor. Instead, multi-receptor activation by the compounds is observed frequently. Nuclear receptor activation by agricultural fungicides from the azole family is not confined to the three receptors that are in focus of this work. The impact of azole fungicides on additional nuclear receptors has also been analyzed in various studies, even though the total amount of available data stays considerably behind the amount of experimental results published for AHR, CAR, and PXR. Just to give some examples, it has been shown that propiconazole, besides exerting effects on the abovementioned receptors, also activates human farnesoid-X-receptor, liver-X-receptor (LXR) α, PPARγ, and RARα in human liver cells, while inhibiting PPARα [16]. The same paper also describes LXRα and PPARα inhibition by tebuconazole [16]. Comparably, imazalil is also an activator of RARα and an inhibitor of PPARα [58]. While exhibiting activity at multiple nuclear receptors, literature data at the same time show that the azole compounds are often only weak to moderate agonists, as compared to model agonists used as reference compounds.

It is tempting to speculate that the potential to simultaneously act on different nuclear receptors could be an important mechanism underlying the biological effects caused by the respective compound in the liver. For example, hepatic steatosis is a complex process that may involve different nuclear receptors, including AHR, CAR, PXR, PPARα, and others, with the nuclear receptor interaction constituting the molecular initiating events in the adverse outcome pathway for liver steatosis, as for example shown in [58]. Interestingly, activation of PXR by a model agonist providing high specificity for the receptor only weakly induces triglyceride accumulation in HepaRG human hepatoma cells, whereas propiconazole and tebuconazole, two much less potent PXR agonists, are much more effective in inducing the accumulation of triglycerides in these cells [16]. Thus, simultaneous activity at multiple receptors triggering different sub-pathways within the steatosis AOP, for example activation of PXR and inhibition of PPARα, possibly play a key role for the high biological activity of azole compounds with respect to triglyceride accumulation. Further research is needed here to elucidate the underlying biochemical mechanisms and to unravel the molecular interplay of the different nuclear receptors.

## 5. Conclusions

The nuclear receptor-activating and/or -inhibiting profiles of azole fungicides are diverse and mostly do not show pronounced compound selectivity for a specific receptor. Instead, multi-receptor agonism and antagonism is observed with individual compounds, with AHR, CAR, and PXR being induced rather than inhibited by most azoles. The often rather weak-to-moderate effects at the receptor level are subject to pronounced species differences, which have been particularly investigated for CAR. Nuclear receptor activation profiles appear suitable to prognosticate many aspects of hepatic effects of azole class compounds. Nonetheless, in vitro nuclear activation assays do not appear to be appropriate for hepatotoxicity predictions as stand-alone tools. Instead, they may be applied as part of an integrated, step-wise testing strategy and to provide mechanistic information useful for risk assessment and especially for the interpretation of inter-species differences.

## Figures and Tables

**Table 1:** Effects of propiconazole on pregnane-X-receptor AHR and AHR targets.

Propiconazole	Mouse		Rat		Human	
AHR	↑	↓	↑	↓	↑	↓
*Cell-free systems*						
Molecular docking					[31]	
*In vitro systems*						
Reporter assay	[25]	[25]			[16,27,31]	
Target mRNA			[5]		[31,37,38]	
Target protein					[26,31]	
Target enzyme activity					[31,35,37]	
*In vivo studies*						
Target mRNA	[6]		[4,31,36]			
Target enzyme activity	[36]		[31,36]			

**Table 2:** Effects of propiconazole on CAR and CAR targets.

Propiconazole	Mouse		Rat		Human	
CAR	↑	↓	↑	↓	↑	↓
*Cell-free systems*						
Molecular docking/FRET					[32]	
*In vitro systems*						
Reporter assay	[33]		[32]		[16,32,33]	
Target mRNA			[32]		[31,32,38]	
Target enzyme activity						[32]
*In vivo studies*						
Target mRNA	[6,20,33,36,39,40]		[5,36]			
Target enzyme activity	[36]		[36]			

**Table 3:** Effects of propiconazole on PXR and PXR targets.

Propiconazole	Mouse		Rat		Human	
PXR	↑	↓	↑	↓	↑	↓
*In vitro systems*						
Reporter assay					[12,16,28,32]	
Target mRNA					[31,32,38]	
Target enzyme activity						[32,35]
*In vivo studies*						
Target mRNA	[6,33,36]		[4,5,36]			

**Table 4:** Effects of epoxiconazole on AHR and AHR targets.

Epoxiconazole	Mouse		Rat		Human	
AHR	↑	↓	↑	↓	↑	↓
*In vitro systems*						
Target mRNA			[64]		[26,63,64]	
Target protein					[26]	
*In vivo studies*						
Target mRNA			[65,66]			
Target enzyme activity			[65,66]			

**Table 5:** Effects of epoxiconazole on CAR and CAR targets.

Epoxiconazole	Mouse		Rat		Human	
CAR	↑	↓	↑	↓	↑	↓
*In vitro systems*						
Reporter assay					[64]	
*In vivo studies*						
Target mRNA	[20,39]		[65,66]			
Target enzyme activity			[65,66]			

**Table 6:** Effects of epoxiconazole on PXR and PXR targets.

Epoxiconazole	Mouse		Rat		Human	
PXR	↑	↓	↑	↓	↑	↓
*In vivo studies*						
Target mRNA			[65,66]			
Target enzyme activity			[65,66]			

**Table 7:** Effects of cyproconazole on AHR and AHR targets.

Cyproconazole	Mouse		Rat		Human	
AHR	↑	↓	↑	↓	↑	↓
*In vitro systems*						
Target mRNA			[64]		[17,64]	
*In vivo studies*						
Target mRNA	[9,10]		[65,66]			
Target protein	[30]					

**Table 8:** Effects of cyproconazole on CAR and CAR targets.

Cyproconazole	Mouse		Rat		Human	
CAR	↑	↓	↑	↓	↑	↓
*In vitro systems*						
Reporter assay					[64]	
Target mRNA					[17]	
*In vivo studies*						
Target mRNA	[9,10,19,20,30,39]		[65,66]			
Target protein	[9,10,19,30]					
Target enzyme activity	[19]		[65,66]			

**Table 9:** Effects of cyproconazole on PXR and PXR targets.

Cyproconazole	Mouse		Rat		Human	
PXR	↑	↓	↑	↓	↑	↓
*In vitro systems*						
Reporter assay					[17]	
Target mRNA					[17]	
Target protein					[17]	
*In vivo studies*						
Target mRNA	[9,10]		[65,66]			
Target enzyme activity	[19]		[65,66]			

**Table 10:** Effects of tebuconazole on AHR and AHR targets.

Tebuconazole	Mouse		Rat		Human	
AHR	↑	↓	↑	↓	↑	↓
*In vitro systems*						
Target mRNA					[26,37,38,63]	
Target protein					[26]	
Target enzyme activity					[35,37]	
*In vivo studies*						
Target mRNA	[10]		[37]			
Target enzyme activity			[37]			

**Table 11:** Effects of tebuconazole on CAR and CAR targets.

Tebuconazole	Mouse		Rat		Human	
CAR	↑	↓	↑	↓	↑	↓
*Cell-free systems*						
Molecular docking/FRET					[32]	
*In vitro systems*						
Reporter assay			[32]			[16,32]
Target mRNA			[32]		[32,37,38]	
Target enzyme activity					[32]	
*In vivo studies*						
Target mRNA	[10]					
Target protein	[9,10]					

**Table 12:** Effects of tebuconazole on PXR and PXR targets.

Tebuconazole	Mouse		Rat		Human	
PXR	↑	↓	↑	↓	↑	↓
*In vitro systems*						
Reporter assay					[16,32]	
Target mRNA					[32,37,38]	
Target enzyme activity					[32]	
*In vivo studies*						
Target mRNA	[10]					

**Table 13:** Effects of prochloraz on AHR and AHR targets.

Prochloraz	Mouse		Rat		Human	
AHR	↑	↓	↑	↓	↑	↓
*In vitro systems*						
Reporter assay					[30,63]	
Target mRNA					[26,63]	
*In vivo studies*						
Target mRNA	[30]		[65,66]			
Target protein	[30]					
Target enzyme activity			[65,66]			

**Table 14:** Effects of prochloraz on CAR and CAR targets.

Prochloraz	Mouse		Rat		Human	
CAR	↑	↓	↑	↓	↑	↓
*In vitro systems*						
Reporter assay					[30]	
*In vivo studies*						
Target mRNA	[30]		[65,66]			
Target protein	[30]					
Target enzyme activity			[65,66]			

**Table 15:** Effects of prochloraz on PXR and PXR targets.

Prochloraz	Mouse		Rat		Human	
PXR	↑	↓	↑	↓	↑	↓
*In vitro systems*						
Reporter assay					[12]	
*In vivo studies*						
Target mRNA			[65]			
Target enzyme activity			[65,66]			

**Table 16:** Effects of imazalil on AHR and AHR targets.

Imazalil	Mouse		Rat		Human	
AHR	↑	↓	↑	↓	↑	↓
*In vitro systems*						
Target mRNA					[26,38,58]	
Target protein					[26]	
Target enzyme activity					[35]	
*In vivo studies*						
Target mRNA	[18]					

**Table 17:** Effects of imazalil on CAR and CAR targets.

Imazalil	Mouse		Rat		Human	
CAR	↑	↓	↑	↓	↑	↓
*In vitro systems*						
Reporter assay					[58]	
Target mRNA					[38,58]	
Target protein					[58]	
*In vivo studies*						
Target mRNA	[18,79]					
Target protein	[18]					

**Table 18:** Effects of imazalil on PXR and PXR targets.

Imazalil	Mouse		Rat		Human	
PXR	↑	↓	↑	↓	↑	↓
*In vitro systems*						
Reporter assay	[79]				[12,58]	
Target mRNA					[38,58]	
Target protein					[58]	
Target enzyme activity						[35]
*In vivo studies*						
Target mRNA	[18,79]					

**Table 19:** Effects of other agricultural fungicides on nuclear receptors.

Compound	AHR	CAR	PXR
Bitertanol	[25]		
Bromuconazole			[34]
Difeconazole	[26]		
Etaconazole		[83]	
Fenbuconazole	[38]	[38]	[12,38]
Flusilazole	[26,63]		
Hexaconazole	[38]	[38]	[38]
Myclobutanil	[5,26,27,38]	[5,20,38]	[5,20,38]
Prothioconazole	[25]		
Thiabendazole			[79]
Triadimefon	[5,6,27]	[5,6,20]	[4,5,6]
Triadimenol	[27]		
Triflumiconazole	[38]	[38]	[38]
Uniconazole	[38]	[38]	[38]

## Supplementary Materials

The following are available online at https://www.mdpi.com/2073-4409/9/5/1192/s1, Table S1: Supplemental Table containing the collection of published azole fungicide-nuclear receptor interactions.
